# Effect of Umbilical Cord Ferritin Level on Auditory Brainstem Response Threshold in Newborns 

**DOI:** 10.22038/ijorl.2019.35305.2163

**Published:** 2020-03

**Authors:** Soumyajit Das, Suvamoy Chakraborty, Chamma Gupta, Rachna Lamichaney, Hafizur Rahman

**Affiliations:** 1 *Department of ENT, Sikkim Manipal Institute of Medical Sciences, Sikkim Manipal University, 5th Mile, Tadong, Gangtok - 737102, Sikkim, India.*; 2 *Department of ENT, Neigrihms, Shillong, India.*; 3 *Department of Biochemistry. Sikkim Manipal Institute of Medical Sciences, Sikkim Manipal University, 5th Mile, Tadong, Gangtok-737102, Sikkim, India.*; 4 *Department of Pathology, Sikkim Manipal Institute of Medical Sciences, Sikkim Manipal University,5th Mile, Tadong, Gangtok- 737102, Sikkim, India.*; 5 *Department of Obstetrics and Gynecology, Sikkim Manipal Institute of Medical Sciences, Sikkim Manipal University,5th Mile, Tadong, Gangtok-737102, Sikkim, India.*

**Keywords:** Amplitude, Evoked response audiometry, Ferritin, Iron, Myelination, New born, Wave latency, Umbilical cord blood

## Abstract

**Introduction:**

Iron plays an important role in myelination. Ferritin is a reliable indicator of the tissue iron store and umbilical cord ferritin level reflects the in utero iron stores. Objective is to study the effect of the umbilical cord ferritin level on the ABR recording in the newborn.

**Materials and Methods:**

The study was conducted in a tertiary care hospital in India with a sample of 250. The study group was divided into Group A (with umbilical cord ferritin level of ≤ 75ng/ml) and Group B (umbilical cord ferritin level > 75ng/ml). Correlation analysis was carried out to study the relation between ferritin level and latency of wave I,III and V. Two sample t test was done between the two groups to study the significance of latency and amplitude of various ABR waves.

**Results:**

There was no correlation between the ferritin and ABR threshold as well as latency and amplitude of ABR waves. A significant prolongation of the absolute latency of wave V and the interwave latency of III – V and I –V of both the ears was found in Group A. The amplitude of the ABR waves did not show any statistical difference between the two groups.

**Conclusion:**

Ferritin levels effect the latency of wave V of ABR and this may be attributed to slow conduction time secondary to altered myelination. Measurement of serum ferritin may be considered as a routine protocol in newborn babies after delivery or before discharge from hospital.

## Introduction

  The prevalence of deafness in South-East Asia ranges from 4.6-8.8% and it is estimated to be around 6.3% (1) in India. The estimated prevalence of adult-onset deafness is 7.6% and that of the childhood-onset deafness is 2% (1). National Sample Survey 58^th^ round (2008) estimated that about 7% of people are born with hearing disability (2). According to the National Family Health Survey 3, the prevalence of anemia in India among women of 15–39 years is 55.3% (3). This rate is higher in the Sikkim, India, where it is measured at 60% (3). Iron deficiency accounts for the majority of anemia cases. Iron is an essential nutrient for the myelination and acts as a co-factor in myelin synthesis. The oligodendrocytes in the brain require a high and constant supply of iron during the process of myelination (4). As such perinatal iron deficiency can affect the myelination process resulting in adverse effects on the central and peripheral nervous system. This may lead to long-lasting behavioral, cognitive, and motor deficits (5,6). Ferritin is a sensitive indicator for the tissue iron stores and the iron depletion starts much before overt anemia sets in. Umbilical cord ferritin concentration is considered a good indicator of fetal iron status during pregnancy (7). The cut-off level of umbilical cord ferritin level is taken to be 75ng/ml as per various studies as this level has been found to be associated with neurodevelopment outcomes in term and premature neonates (8,7). The present study aimed to investigate the relationship of cord blood ferritin level with auditory brainstem response (ABR) threshold of the newborns. In addition, this study involves the investigation of the effect of cord blood ferritin level on latency and amplitude of ABR waves.

## Materials and Methods

The present study was conducted in a tertiary care hospital in a northeastern state of India with a sample size of 250 neonates. The ethical clearance for the current study was obtained from the Institutional Ethical Committee. The inclusion criteria entailed: 1) parental consent and 2) new born delivered out of term pregnancy. 

The exclusion criteria included: 1) parents who did not agree to participate in the study, 2) newborns with known risk factors for hearing loss (e.g., septicemia, neonatal hyper- bilirubinemia congenital malformations, maternal hypothyroidism), low birth weight, prematurity and low Apgar score at birth, and history of ototoxic drugs or alcohol abuse by the mother during pregnancy. Umbilical cord blood sampling was performed after severing of the umbilical cord from the placenta and approximately 4ml of cord blood was collected with a sterile syringe from the umbilical vein in a gel vacutainer. The sample was centrifuged to separate the serum and estimation of the ferritin level was conducted by the solid phase enzyme-linked immunosorbent assay in a VIDAS (Biomerieux) fully automated immunoassay. All serum estimation was carried out after the standard calibration of the equipment.

 The ABR recording was performed within 48-72 h after birth in the audiology section of the department. In addition, the Interacoustics Eclipse ABR machine was used for this purpose. The audiologist who performed the ABR test was unaware of the cord ferritin level of the newborns. The electrode placement was performed with a vertex active electrode and ipsilateral mastoid as the reference electrode while the ground electrode was placed over the cheek. The stimulus was click stimulus with a rate of 33.1/sec in rarefaction polarity with an interelectrode impedance of 5kOhm. The used electrodes were gold cup electrodes and electrode placement was performed after adequate skin preparation with a Nuprep skin preparation gel. A low-pass filter of 30Hz and a high-pass filter of 1500Hz were used. Moreover, a minimum of 2000 clicks was recorded to obtain the best tracings. The ABR threshold was regarded as the lowest intensity of the click stimulus where a definite and recognizable wave V morphology could be detected. The latency values and amplitude of the waves were recorded at 70dB intensity levels in all the newborns. The recording was performed during the natural sleep or resting condition with the infant lying supine and flat on a couch. An insert ear probe tip was used for the provision of the sound stimulus. The ABR recording was carried out for all the 250 samples. However, ABR tracings in 11 neonates were inconclusive and it was not possible to obtain definitive interpretation of wave morphology and latency. Consequently, these 11 recordings and their corresponding blood samples were excluded from the final analysis. The final analysis was performed on 239 remaining samples. The Pearson’s correlation analysis was carried out between the ferritin level and various ABR parameters, including absolute latencies of waves V, III, I, and inter-wave latencies of waves I-V, I-III, and III-V of both ears. The ferritin levels were further divided into two groups based on the cut-off level of 75ng/ml from previous studies (6). Those with ferritin values≤75ng/ml were designated as group A and those with ferritin level>75ng/ml were designated as group B. The total sample size of group A was 36 while that of group B was 203. A two-sample t-test was carried out between the two groups to determine any statistical difference between the ABR parameters of the two groups. Statistical analysis was performed with MS Excel (Microsoft, 2007). The confidence limit of the test was fixed at 95% and a p-value of less than 0.05 was considered statistically significant.

## Results

**Fig 1:**
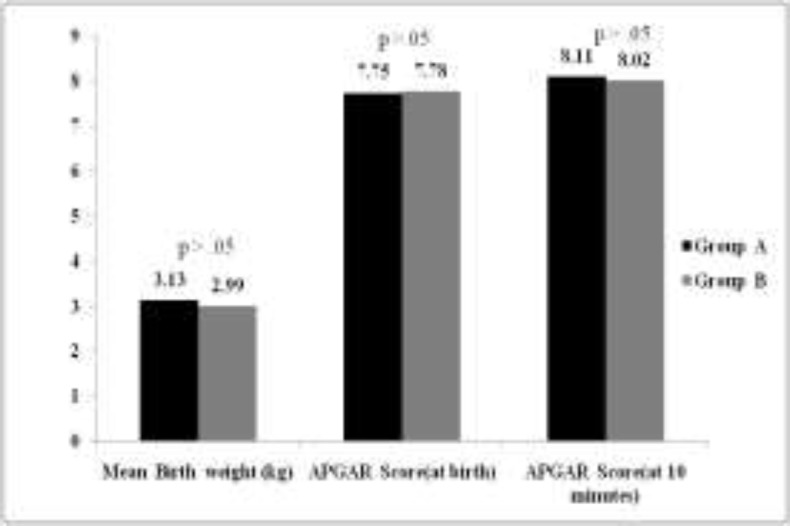
Birth weight, APGAR score ( at birth and at 10 minutes) between the two groups

A total of 239 neonates were selected for the final analysis of which 118 neonates were boys and 121 girls. The mean gestational age of the neonates were 38.8 weeks. The average birth weight and the Apgar score (at birth and at 10 min) of the neonates in the two groups were similar and no statistically significant difference was detected (Fig.1). In addition, no significant difference was observed in the min ABR threshold among the two groups (groups A and B).The ferritin level did not show any correlation with the latency values of waves I, III, and V and the interlatency values of waves I-V, I–III, and III-V. 

The latency value of wave V of both ears was significantly prolonged in group A, compared to group B (P<0.05 for both left and right ears). The latency of waves I and III of both ears showed no significant difference between the two groups. It was also observed that the interlatency of waves I–V was prolonged in group A, as compared with group B for both ears (P<0.05). A significant prolongation of interwave latencies of III-V for both ears was noted in group A as compared to group B. However, there was no difference in the interlatencies of I–III between the two groups (Table.1). 

In addition, there was no significant difference in the latency of wave V among the male and female newborns. The amplitude of waves I, III, and V revealed no significant difference between the two groups (P>0.05).

**Table 1:** Results of absolute latency, interwave latency and amplitude and average threshold of ABR waves

**BERA Parameters**	**GROUP A n=36**		**GROUP B n=203**		**P value**	
Mean of Latency (ms)	Right ear	Left ear	Right ear	Left Ear	Right ear	Left Ear
Wave I	1.86	1.87	1.85	1.88	0.42	0.45
Wave III	4.4	4.38	4.35	4.33	0.29	0.32
Wave V	6.46	6.46	6.24	6.24	0.02	0.02
Mean of Interwave Latency (ms)						
I – III	2.51	2.53	2.50	2.42	0.45	0.16
I – V	4.61	4.57	4.40	4.34	0.04	0.03
III – V	2.09	2.08	1.90	1.91	0.02	0.02
Mean of Amplitude (µV)						
Wave I	0.13	0.12	0.13	0.13	0.45	0.33
Wave III	0.14	0.14	0.16	0.17	0.31	0.23
Wave V	0.29	0.21	0.28	0.27	0.41	0.06
Average ABR threshold (dB)	30	30	30.09	30.04	0.07	0.15

## Discussion

Iron plays a major role in myelination as it is a co-factor and integral component of several enzymes (5,6,8), as well as it is involved in the functioning of neurotransmitters (e.g., gamma-aminobutyric acid) (9,10). Altered myelination secondary to perinatal iron deficiency can lead to long-lasting behavioral, cognitive, motor, and language deficits despite subsequent iron therapy (11,2). Iron deficiency in guinea pigs during pregnancy and lactation demonstrated an elevated ABR threshold in the subsequent progeny. It was found to affect all frequencies suggesting that all parts of the cochlea were affected (13). Ferritin is a sensitive indicator of tissue iron stores. The major function of ferritin is to provide iron storage which can be utilized for required heme synthesis. Iron stores in the central nervous system deplete before there is a change in the red cell production (14). As such tissue, iron deficiency before the onset of anemia can have adverse neurodevelopment outcomes as it disrupts the normal development of the auditory pathway and results in altered conduction velocity (15). The ABR is an important tool for assessing the brainstem auditory pathway. The interpeak latencies between the waves indirectly measure the neural conduction in the auditory pathway and are used clinically to detect various subclinical lesions of the auditory nerve, including demyelinating disease. 

In utero iron status assessed by the umbilical cord ferritin level strongly influences auditory neural development (7). Moreover, findings of a study conducted by Sanjiv Amin et al.(7) showed that infants with latent iron deficiency had significantly prolonged absolute latencies for waves III and V in ABR, compared to infants with normal iron levels. Furthermore, Shankar et al. (16) in their study established a correlation between the ABR responses and haematological parameters of children with iron deficiency. Their study found a definite correlation between the severity of anemia and the degree of neuro-physiological deficits (16). A similar finding was also reported by ElAlfy et al., as they found prolonged interpeak latencies among the neonates with latent iron deficiency (17). Pallone et al. delineated similar findings in a recent study conducted in Brazil where significantly higher wave V and interwave latency of ABR waves were detected among the newborns with latent iron deficiency (18). In addition, Algarin et al. revealed similar prolongation of absolute latencies of the ABR waves in children with iron deciciency (19). Moreover, the present study pointed towards prolonged auditory conduction as is denoted by the prolongation of the absolute latency of wave V in the neonates with low ferritin levels. 

There was no prolongation of the absolute latencies of waves I and III in the current study. The present study found a significant prolongation of the interwave latencies of III–V and I-V between the two groups. However, there was no prolongation of the interwave latencies from I–III. The normal latencies of waves I and III as observed in the present study were not in line with the studies conducted by ElAlfy et al. and Pallone et al. Although a definite reason for this discrepancy could not be ascertained, yet it may be possible that this deviation between the studies be due to the different period of exposure of the fetus and development of auditory pathway to deplete iron stores. The prolongation of interwave latencies of I–V and waves III–V may be due to the prolonged central conduction time which occurs due to altered myelination. It has been suggested that altered myelination affects the late ABR components (interwave latencies III–V) more than I–III interwave latencies due to the “centripetal” progression of the myelination process (20). Myelination of the auditory pathway occurs within 26-29 weeks from the proximal end of the cochlear nerve (21). 

However, the central auditory pathway continues with the process of myelination until the second post-natal life (22). Therefore, the brain iron deficiency during this critical period may represent long-lasting defects in the ABR latency. The present study did not attempt to examine anemia or its severity and relation to the ABR threshold. Moreover, the current study found no correlation between the latencies of waves I, III, V, and ferritin. In addition, there was no significant difference in the minimum ABR threshold between the two groups.

The amplitude of the ABR waves is not widely used in clinical practice and most studies have focused on the latency of the ABR waves. There have been contradictory results on the changes in amplitude of the ABR in patients with speech and language disorders (23). Moreover, the effect of iron deficiency anemia on the amplitude of ABR waves is not established. Jougleux et al. (24) in their study found an increase in the amplitude of the ABR waves among the guinea pigs with iron deficiency. Studies conducted on humans failed to find any changes in the amplitude of ABR waves (19,20); however, only one study carried out by Shanker et al. showed low amplitude of the ABR waves in children with iron deficiency (16). The limitation of the present study was that it did not take into account the maternal iron stores during the pregnancy. 

As a result, it was not possible to comment on the status of the fetal iron stores at a specific period of the intrauterine life. In the present study, the authors considered a single ABR recording. Consequently, it was not possible to comment on whether the prolongation between the two groups was temporary or permanent. However, the findings of the current study suggest that it may be worthwhile to assess the ferritin level in the newborns at birth or at the time of hospital discharge. This may help the physicians to identify the newborns at-risk of adverse neurodevelopment outcomes; therefore, providing necessary and timely interventions. 

## Conclusion

The present study showed that low ferritin level causes prolongation in the latency of the wave V of ABR in newborns and affects central conduction of the waves. It is noteworthy to study whether the prolongation of latency among the low ferritin group is temporary or permanent. There are exciting avenues for further research in this field in studying and identifying the period of maximum vulnerability of the fetus to low iron stores during the intrauterine life. Further longitudinal studies are warranted to determine long-term clinical implications of the prolonged latency noted among the low ferritin group.

## Funding’s

The present study was funded by the Indian Council for Medical Research (ICMR) under the seed grant scheme vide IRIS cell no 2014 -3122, File No: 5/7/1277/2015-RCH.
